# Endothelial Dysfunction and Cardiovascular Disease: History and Analysis of the Clinical Utility of the Relationship

**DOI:** 10.3390/biomedicines9060699

**Published:** 2021-06-20

**Authors:** Peter J. Little, Christopher D. Askew, Suowen Xu, Danielle Kamato

**Affiliations:** 1 Sunshine Coast Health Institute, School of Health and Behavioural Sciences, University of the Sunshine Coast, Birtinya, QLD 4575, Australia; caskew@usc.edu.au; 2 Department of Pharmacy, Xinhua College, Sun Yat-sen University, Tianhe District, Guangzhou 510520, China; d.kamato@uq.edu.au; 3 Pharmacy Australia Centre of Excellence, School of Pharmacy, The University of Queensland, Woolloongabba, QLD 4102, Australia; 4 VasoActive Research Group, School of Health and Behavioural Sciences, University of the Sunshine Coast, Sippy Downs, QLD 4556, Australia; 5 Department of Endocrinology and Metabolism, Division of Life Sciences and Medicine, First Affiliated Hospital of USTC, University of Science and Technology, Hefei 230037, China; sxu1984@ustc.edu.cn

**Keywords:** endothelium, atherosclerosis, complications, nitric oxide, endothelin, plethysmography

## Abstract

The endothelium is the single-cell monolayer that lines the entire vasculature. The endothelium has a barrier function to separate blood from organs and tissues but also has an increasingly appreciated role in anti-coagulation, vascular senescence, endocrine secretion, suppression of inflammation and beyond. In modern times, endothelial cells have been identified as the source of major endocrine and vaso-regulatory factors principally the dissolved lipophilic vosodilating gas, nitric oxide and the potent vascular constricting G protein receptor agonists, the peptide endothelin. The role of the endothelium can be conveniently conceptualized. Continued investigations of the mechanism of endothelial dysfunction will lead to novel therapies for cardiovascular disease. In this review, we discuss the impact of endothelial dysfunction on cardiovascular disease and assess the clinical relevance of endothelial dysfunction.

## 1. Introduction

The defining characteristics of biological systems are a set of functional organs and hemodynamic systems that facilitate the functioning of organs. A major feature is the manner in which these compartments are separated and in mammalian systems, this is via the single cell lining of blood vessels known as the endothelium [1,2,3,4]. Although initially recognized for its barrier function, the understanding of the role of the endothelium has expanded greatly in the last few decades [2,5]. In its original manifestation, the role of the endothelium was determined to facilitate the bilateral interchange of nutritional and waste materials between the blood and the tissues and critically to control clotting of the blood by routinely preventing clot formation and restricting clot formation to the healing of injured tissues. In modern times, the endothelium has been recognized as the source of a variety of highly important biological mediators some of which have been amongst the most important findings in the history of biology [6,7,8]. These mediators are vasoconstrictors, vasodilators and regulators of thrombosis and inflammation. In recent times, the interest in inflammation in pathology and pathophysiology has increased enormously and the endothelium has been identified as a key mediator in the regulation of inflammation [9,10,11]. The role of the endothelium in diseases and its potential as a therapeutic target is currently of major interest especially in cardiovascular disease and in the metastasis of cancer [9,12,13,14]. Injury to endothelial cells or more broadly disturbance of the homeostasis in the endothelium is termed “endothelial dysfunction”—this term originally related to the reduced vasodilatory capacity but has expanded with the evolution of the understanding of the role of the endothelium in the chronic inflammation of various diseases [1,9,11,15,16,17,18].

## 2. History of Endothelial Dysfunction

The endothelium and endothelial cells have been the source of some of the major discoveries in human biology. The phenomenon of “endothelial dysfunction” has been in the literature for at least 25 years. Endothelial dysfunction (ED) can be narrowly defined as the vasoactive property or reduced vasodilatory capacity and more broadly as any changes that impact the vasoprotective homeostatic function of the endothelium. Endothelial cells were found to be the source of vasoconstricting 21 amino acid peptides known as endothelins which were discovered in 1987 by the Japanese group of Masaki and colleagues who were subsequently awarded the prestigious Tsukuba Prize [7]. The other mechanistically contrasting factor was a vasodilator and this was the discovery of the dissolved lipophilic gas, nitric oxide (NO), as the factor designated for many years as Endothelial-Derived Relaxing Factor (EDRF) [6,19,20,21,22]. The discovery that EDRF was a dissolved gas and that it could mediate a traditional signaling pathway, activation of cyclic GMP kinase, was a great surprise to the scientific community as many had expected EDRF to follow on from the discovery of endothelin and to be a peptide. Furchgott, Ignarro and Murad received the Nobel Prize in Physiology or Medicine in 1998 for the discovery of EDRF being NO and its signaling properties. NO mediates vasodilation and numerous other biochemical and cellular responses that are protective of the endothelium and underlying tissues.

## 3. Endothelial Dysfunction and Cardiovascular Disease

Several clinical studies speculate that ED leads to accelerated atherosclerosis. The two driving forces of ED that lead to atherosclerosis include the impact on vaso-regulation and chronic unresolving inflammation. In response to vascular injury, a plethora of pro-inflammatory cytokines and chemokines are released. Inflammatory cascades are complex involving pro- and anti-inflammatory molecules and cells. We previously described the multiple possible inflammatory targets, which represent therapeutic targets in this area [23,24].

In this review, we referred to the two aspects of ED—the narrow application related to vasodilatation and the broader context of inflammation. The functional and therapeutic difference in these two designations are exemplified by the actions of newer anti-diabetes drugs—these drugs including sodium-glucose transport protein 2 (SGLT2) inhibitors and glucagon-like peptide-1 (GLP-1) agonists have beneficial effects in large clinical trials where cardiovascular events and deaths are reduced by treatment with the index agents. It has however been very difficult to show consistent favorable actions on endothelial function assessed as enhanced vasodilation [25,26]. This suggests that the favorable cardiovascular effects arise from anti-inflammatory actions on the endothelium; these anti-inflammatory actions are more disparate and difficult to characterize than measures of vasodilatory capacity.

One area that very explicitly demonstrates the role of the endothelium is that of erectile function and dysfunction [27,28]. Erectile dysfunction usually precedes cardiovascular disease and might be seen as an early marker of clinically relevant cardiovascular disease. It is perhaps not intuitive that proper functioning involves vasodilatation as opposed to vasoconstriction. Vasodilatation leads to enhanced blood flow and the necessary biological response [29]. Clearly, ED, which reduces vasodilatation, inhibits the process of erectile function. It is highly instructive in understanding the perniciousness of cardiovascular disease that erectile dysfunction correlates with coronary artery disease [28]. This occurs because the development of cardiovascular disease occurs throughout the vascular tree but its manifestation varies greatly from vascular bed to vascular bed and from individual to individual [30]. In this context, erectile dysfunction serves as an indicator of cardiovascular disease and indicates the need for monitoring and assessment of the status of vascular beds associated with heart disease, strokes and lower limb amputations.

A normal endothelium clearly regulates the biologically desirable quiescence of the vasculature and protects against atherosclerosis. The effects on the endothelium can be a very difficult area to study in vitro, especially in the context of blood pressure or inflammation. In vitro data typically arise from studies on isolated vascular cells, however, the impact of other factors such as NO, endothelin (ET)-1 and angiotensin (Ang)-II has multiple actions in driving the development of atherosclerosis. The role of the immune system and inflammation is very complex involving multiple biochemical and cellular mediators and, although the knowledge on the immune system is rapidly evolving, more insights are required before an amenable therapeutic intervention can be described. Human trials of anti-inflammatory strategies such as the use of anti-interleukin (IL)-1β antibodies in the CANTOS trial have shown promising results but the initial data arise in high-risk patients and secondary cardiovascular disease rather than the desirable targets occurring in younger people with early atherosclerosis [31,32].

SGLT2 inhibitors (SGLT2i) block the renal reabsorption of glucose and have a major effect on increasing glucose excretion and decreasing hyperglycaemia. SGLT2i reduce cardiovascular events and death in clinical trials [33]. Emerging data show that SGLT2i, somewhat surprisingly, have a multitude of favourable actions on the cardiovascular system to underwrite their actions in preventing events in clinical trials. The favourable effects of empagliflozin on cardiovascular events [33,34] were followed up by a specific study of its effects on ED assessed by the reactive hyperaemia peripheral arterial tonometry index (RHI) in a multicentre double-blind clinical trial in 16 centres and covering over 100 patients given empagliflozin or placebo for 24 weeks (EMBLEM trial) [35]. There was no difference in RHI in patients given either empagliflozin or placebo [35]. This study looked at the narrow definition of ED and it may well be that the clinical trial outcomes result from the studies mentioned above are due to the broader anti-inflammatory actions on the endothelium. In pre-clinical studies, dapagliflozin improved ED in a mice model of type 2 diabetes and also altered the gut microbiome which is an emerging determinant of ED and cardiovascular disease that needs to be followed up.

## 4. Endothelial Dysfunction and Mechanisms of Atherosclerosis

The critical factor in this area is the relationship between ED and the initiation, development, progression and clinical manifestation being the rupture of an atherosclerotic plaque and a heart attack or stroke [36,37]. There are many examples of this relationship being assumed or implied, perhaps beyond what the present data allow. This is especially prescient in that the actual mechanism(s) of atherogenesis remains incompletely understood and the ranking therapies are those that target risk factors, a blunt approach, rather than a specific molecular or cellular mechanism [9,38,39]. The very early concepts expressed in the “response to injury” hypothesis proposed that physical damage to the endothelium, with consequential disruption of the barrier function, led to the commencement of atherosclerosis [8,40,41]. However, careful studies failed to demonstrate actual physical damage and the hypothesis was advanced to relate to the biochemical “damage” implicit in the activation of endothelial cells in ED and their role in inflammation. This and other proposed mechanisms of atherosclerosis remain under constant investigation.

One of the specific mechanisms that shows some promise is the role of the sub-endothelial retention of atherogenic lipoproteins being the initiating step in atherogenesis [42,43,44]. This is known as the “response to retention” hypothesis of atherosclerosis and it is supported by molecular, cellular, animal studies and some compelling human pathological data [45,46,47,48,49]. Modified glycosaminoglycan (GAG) chains on proteoglycans, notably biglycan [42], attract and retain all passing cholesterol within the vessel wall. We demonstrated that hormones and growth factors released from endothelial cells (e.g., ET1 and AngII) lead to the modification of the GAG chain on proteoglycans [50,51]. We originally proposed that this response was a post-inflammatory response but having recently shown that lipopolysaccharides (LPS) stimulate GAG elongation, we now propose GAG elongation as one of the earliest steps and possibly a useful marker and predictor of inflammation in tissues [52]. We observed a highly specific signalling pathway that regulates GAG chain elongation [50]. Growth factors, hormones and endotoxins acting via completely different receptor pathways signal specifically via the linker region of Smad2 transcription factor to regulate the genes associated with GAG chain modification [52,53,54,55,56,57,58]. Furthermore, animal studies provide proof of principle that treating atherosclerosis-prone mice fed a high-fat diet an inhibitor of GAG elongation can prevent at least 50 percent of the lipid deposition [46,59]. More recent data show that the component of lipid deposition in the vessel wall which is not inhibited by statin treatment can be completely prevented by the co-administration of a GAG elongation inhibitor (Afroz, Kamato and Little, unpublished observations).

## 5. Endothelial Dysfunction—Clinical Aspects

**Figure 1:**
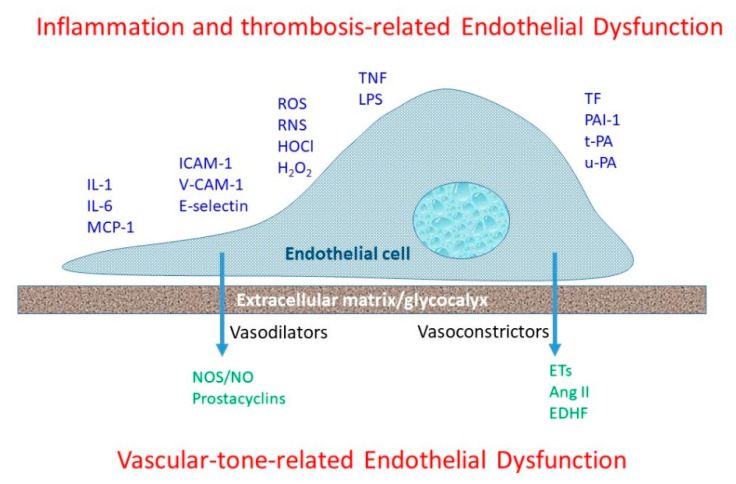
Mediators of endothelial dysfunction and endothelium-derived factors that influence the development and progression of atherosclerosis. Cardiovascular risk factors render the endothelium susceptible to the generation of atherosclerosis. Endothelial dysfunction affects vascular reactivity. Cytokines, inflammatory mediators, reactive oxygen species, and pro-thrombotic factors draw endothelial cells into thrombotic and immune responses. Under pathophysiological conditions, endothelial cells release hormones, cytokines and growth factors that impact vascular contractility.

Clinical evaluation of ED corresponds to two expressions, impaired vasoactive regulation and inflammation and thrombotic response [18] (Figure 1). While ED is characterized pathophysiologically by a state that is prothrombiotic, proimflammatory and atherogenic [60], the assessment of ED has mainly focused on the vasodilatory function of the artery and the ability to regulate tone, resistance and blood flow. Early assessments of coronary artery endothelial function in humans relied on the direct intracoronary artery infusion of endothelium-dependent vasodilators (such as acetylcholine) accompanied by quantitative coronary artery angiography [61,62]. This same method is used in the peripheral and coronary circulations [63], and arguably the hyperemia or dilation response that is induced by the direct infusion of acetylcholine represents the criterion or gold-standard measure of endothelial function. However, the invasive nature of this approach, including the need for antra-arterial catheterization and medical supervision, limits its utility in research and clinical settings. With this, several non-invasive methods have been developed, whereby artery shear–stress is manipulated to induce a corresponding increase in blood flow and/or artery diameter that is, at least in part, endothelial-dependent.

Flow-mediated dilation (FMD) of the brachial artery is a frequently used method in clinical research that was introduced as a non-invasive measure of endothelial function in 1992 by Celemejer et al. [64]. While the participant is rested and lying in a supine position, a forearm blood pressure cuff is inflated for 5 min, and then released to induce local reactive hyperemia and shear stress. Ultrasound imaging is used to measure the diameter of the brachial artery at baseline and during the period following cuff-release, and the difference between these measures represents FMD. This dilation response is attenuated when the NO-inhibitor L-NMMA is infused locally, which provides some confirmation of the NO-dependence of FMD [65,66,67]. Consistent with the notion that ED is involved in the initiation of atherosclerosis, impaired FMD has been reported as an early biomarker of the development of atherosclerotic disease, and is associated with the progression of carotid–intima thickening [68,69]. Moreover, FMD is impaired in groups of patients known to be at a high risk of cardiovascular events [70,71]; indeed, FMD is negatively associated with the risk of cardiovascular events and mortality in those with and without established cardiovascular disease [72,73]. When FMD is assessed using the recommended standardized method, a 1-point increase in FMD (e.g., 5 to 6% dilation) is associated with a 9% (95% CI: 4% to 13%) decrease in the future risk of cardiovascular events [73].

Despite its widespread use in research, FMD has not been adopted in routine practice as a clinical assessment tool. This is likely due to the technical requirements of FMD measurement, including access to suitable ultrasound devices and well-trained sonographers, as well as the time and expertise required for image analysis. Alternative methods that may be better suited to clinical settings have been developed and tested, including passive leg-movement hyperaemia [74,75], which also relies on the use of ultrasound, and reactive hyperaemia, which can be readily measured at the limbs or digits using plethysmography or arterial tonometry. The Endo-PAT device provides an automated measure of reactive hyperaemia at the fingers and is reported to be the only FDA-approved device for the noninvasive assessment of endothelial function [76]. Interestingly, measures derived from the Endo-PAT are only modestly correlated with FMD, and each method differs in its association with classical risk factors. This likely reflects differences in the vasculature being assessed, where FMD is a measure of macrovascular function and endo-PAT reflects microvascular function, and may also reflect different pathologies [77].

Many traditional risk factors only account for around 50–60 percent of cardiovascular events [78]. The uptake of any measure of endothelial function in clinical practice has the potential to fill this “risk factor gap” and add value to disease classification and prognosis. To this end, an understanding of the natural variance of endothelial function is needed. Using standardized assessment methods, age- and sex-specific reference values for brachial artery FMD were recently published which provides a normative reference for future research, and potentially for clinical practice [79].

## 6. Conclusions

In conclusion, the study of ED has provided great insights into the initiation and progression of cardiovascular disease in patients. Many of the data from cardiovascular disease clinical trials can be explained by actions of drugs, directly or indirectly through their actions on risk factors, by their actions on the endothelium. Although the original concepts focused mostly on the role of the endothelium in vascular tone and the regulation of blood flow and pressure, it has more recently been recognized that the endothelium has a profound role in inflammation which underlies many disease states. The expression of pro-inflammatory molecules on the surface of endothelial cells and the secretion of pro-inflammatory, pro-atherosclerotic and pro-thrombotic substances indicate a role of the endothelium beyond its barrier function and role in vascular tone. It is likely that studies of endothelial function will become more common in the clinic and provide early insights into the development of atherosclerosis in patients also leading to the basis of early interventions to treat cardiovascular risk factors which will prevent or delay the occurrence of cardiovascular events in susceptible patients.

The clinical assessment of ED may be useful in some circumstances, but as the mechanistic links between ED and atherosclerosis remain mostly speculative, this does not directly point to a mechanism-based therapeutic intervention and treatment remains empirical. Treatment to prevent atherosclerosis and cardiovascular disease should focus on traditional established cardiovascular risk factors whilst continuing studies delve into the mechanism of ED and the relationship between ED and the initiation and progression of atherosclerosis.

## References

[1] Gimbrone M.A. (1980). Endothelial Dysfunction and the Pathogenesis of Atherosclerosis. Atherosclerosis, V.

[2] Gimbrone M.A., García-Cardeña G. (2016). Endothelial cell dysfunction and the pathobiology of atherosclerosis. Circ. Res..

[3] Sade R.M., Folkman J. (1972). En face stripping of vascular endothelium. Microvasc. Res..

[4] Weibel E.R., Palade G.E. (1964). New Cytoplasmic Components in Arterial Endothelia. J. Cell Biol..

[5] Topper J.N., Gimbrone M.A. (1999). Blood flow and vascular gene expression: Fluid shear stress as a modulator of endothelial phenotype. Mol. Med. Today.

[6] Furchgott R.F. (1996). The Discovery of Endothelium-Derived Relaxing Factor and Its Importance in the Identification of Nitric Oxide. JAMA.

[7] Yanagisawa M., Kurihara H., Kimura S., Tomobe Y., Kobayashi M., Mitsui Y., Yazaki Y., Goto K., Masaki T. (1988). A novel potent vasoconstrictor peptide produced by vascular endothelial cells. Nature.

[8] Ross R. (1986). The pathogenesis of atherosclerosis. N. Engl. J. Med..

[9] Libby P., Aikawa M., Jain M. (2006). Vascular endothelium and atherosclerosis. The Vascular Endothelium II.

[10] Williams S.L., Milne I.R., Bagley C.J., Gamble J.R., Vadas M.A., Pitson S.M., Khew-Goodall Y. (2010). A proinflammatory role for proteolytically cleaved annexin A1 in neutrophil transendothelial migration. J. Immunol..

[11] Vadas M.A., Gamble J.R. (1990). Regulation of the adhesion of neutrophils to endothelium. Biochem. Pharmacol..

[12] Libby P., Ridker P.M., Maseri A. (2002). Inflammation and atherosclerosis. Circulation.

[13] Browder T., Butterfield C.E., Kraling B.M., Shi B., Marshall B., O’Reilly M.S., Folkman J. (2000). Antiangiogenic scheduling of chemotherapy improves efficacy against experimental drug-resistant cancer. Cancer Res..

[14] Browder T., Folkman J., Pirie-Shepherd S. (2000). The hemostatic system as a regulator of angiogenesis. J. Biol. Chem..

[15] Anderson T.J., Gerhard M.D., Meredith I.T., Charbonneau F., Delagrange D., Creager M.A., Selwyn A.P., Ganz P. (1995). Systemic nature of endothelial dysfunction in atherosclerosis. Am. J. Cardiol..

[16] Laurent S., Vanhoutte P., Cavero I., Chabrier P.E., Dupuis B., Elghozi J.L., Hamon G., Janiak P., Juillet Y., Kher A. (1996). The arterial wall: A new pharmacological and therapeutic target. Fundam. Clin. Pharmacol..

[17] Libby P. (2002). Inflammation in atherosclerosis. Nature.

[18] Clapp B.R., Hirschfield G.M., Storry C., Gallimore J.R., Stidwill R.P., Singer M., Deanfield J.E., MacAllister R.J., Pepys M.B., Vallance P. (2005). Inflammation and endothelial function: Direct vascular effects of human C-reactive protein on nitric oxide bioavailability. Circulation.

[19] Furchgott R., Zawadzki J. (1980). The obligatory role of endothelial cells in the relaxation of arterial smooth muscle by acetylcholine. Nature.

[20] Palmer R.M., Ferrige A., Moncada S. (1987). Nitric oxide release accounts for the biological activity of endothelium-derived relaxing factor. Nature.

[21] Ignarro L.J., Buga G.M., Wood K.S., Byrns R.E., Chaudhuri G. (1987). Endothelium-derived relaxing factor produced and released from artery and vein is nitric oxide. Proc. Natl. Acad. Sci. USA.

[22] Rapoport R.M., Murad F. (1983). Agonist-induced endothelium-dependent relaxation in rat thoracic aorta may be mediated through cGMP. Circ. Res..

[23] Little P.J., Chait A., Bobik A. (2011). Cellular and cytokine-based inflammatory processes as novel therapeutic targets for the prevention and treatment of atherosclerosis. Pharmacol. Ther..

[24] Xu S., Ilyas I., Little P.J., Li H., Kamato D., Zheng X., Luo S., Li Z., Liu P., Han J. (2021). Endothelial Dysfunction in Atherosclerotic Cardiovascular Diseases and Beyond: From Mechanism to Pharmacotherapies. Pharmacol. Rev..

[25] Ma X., Liu Z., Ilyas I., Little P.J., Kamato D., Sahebka A., Chen Z., Luo S., Zheng X., Weng J. (2021). GLP-1 receptor agonists (GLP-1RAs): Cardiovascular actions and therapeutic potential. Int. J. Biol. Sci..

[26] Liu Z., Ma X., Ilyas I., Zheng X., Luo S., Little P.J., Kamato D., Sahebkar A., Wu W., Weng J. (2021). Impact of sodium glucose cotransporter 2 (SGLT2) inhibitors on atherosclerosis: From pharmacology to pre-clinical and clinical therapeutics. Theranostics.

[27] Bocchio M., Desideri G., Scarpelli P., Necozione S., Properzi G., Spartera C., Francavilla F., Ferri C., Francavilla S. (2004). Endothelial cell activation in men with erectile dysfunction without cardiovascular risk factors and overt vascular damage. J. Urol..

[28] Gandaglia G., Briganti A., Jackson G., Kloner R.A., Montorsi F., Montorsi P., Vlachopoulos C. (2014). A systematic review of the association between erectile dysfunction and cardiovascular disease. Eur. Urol..

[29] Cartledge J., Minhas S., Eardley I. (2001). The role of nitric oxide in penile erection. Expert Opin. Pharmacother..

[30] Weng W., Kong S.X., Ganguly R., Hersloev M., Brett J., Hobbs T., Baeres F.M.M. (2020). The prevalence of cardiovascular disease by vascular bed and impact on healthcare costs in a large, real-world population with type 2 diabetes. Endocrinol. Diabetes Metab..

[31] Ibanez B., Fuster V. (2017). CANTOS: A Gigantic Proof-of-Concept Trial. Circ. Res..

[32] Ridker P.M. (2017). Mortality Differences Associated with Treatment Responses in CANTOS and FOURIER: Insights and Implications. Circulation.

[33] Zinman B., Wanner C., Lachin J.M., Fitchett D., Bluhmki E., Hantel S., Mattheus M., Devins T., Johansen O.E., Woerle H.J. (2015). Empagliflozin, cardiovascular outcomes, and mortality in type 2 diabetes. N. Engl. J. Med..

[34] Lee S. (2017). Update on SGLT2 Inhibitors-New Data Released at the American Diabetes Association. Crit. Pathw. Cardiol..

[35] Tanaka A., Shimabukuro M., Machii N., Teragawa H., Okada Y., Shima K.R., Takamura T., Taguchi I., Hisauchi I., Toyoda S. (2019). Effect of Empagliflozin on Endothelial Function in Patients with Type 2 Diabetes and Cardiovascular Disease: Results from the Multicenter, Randomized, Placebo-Controlled, Double-Blind EMBLEM Trial. Diabetes Care.

[36] Falk E. (1989). Morphologic features of unstable atherothrombotic plaques underlying acute coronary syndromes. Am. J. Cardiol..

[37] Falk E., Fernandez-Ortiz A. (1995). Role of thrombosis in atherosclerosis and its complications. Am. J. Cardiol..

[38] Nigro J., Osman N., Dart A.M., Little P.J. (2006). Insulin Resistance and Atherosclerosis. Endocr. Rev..

[39] Ross R. (1999). Atherosclerosis—An inflammatory disease. N. Engl. J. Med..

[40] Ross R., Glomset J.A. (1976). The pathogenesis of atherosclerosis (second of two parts). N. Engl. J. Med..

[41] Ross R., Glomset J.A. (1976). The pathogenesis of atherosclerosis (first of two parts). N. Engl. J. Med..

[42] Williams K.J., Tabas I. (1995). The response-to-retention hypothesis of early atherogenesis. Arterioscler. Thromb. Vasc. Biol..

[43] Williams K.J., Tabas I. (1998). The response-to-retention hypothesis of atherogenesis reinforced. Curr. Opin. Lipidol..

[44] Little P.J., Osman N., O’Brien K.D. (2008). Hyperelongated biglycan: The surreptitious initiator of atherosclerosis. Curr. Opin. Lipidol..

[45] Nakashima Y., Fujii H., Sumiyoshi S., Wight T.N., Sueishi K. (2007). Early human atherosclerosis: Accumulation of lipid and proteoglycans in intimal thickenings followed by macrophage infiltration. Arterioscler. Thromb. Vasc. Biol..

[46] Ballinger M.L., Osman N., Hashimura K., de Hann J., Jandeleit-Dahm K., Allen T.J., Tannock L.R., Rutledge J.C., Little P.J. (2010). Imatinib inhibits vascular smooth muscle proteoglycan synthesis and reduces LDL binding in vitro and aortic lipid deposition in vivo. J. Cell Mol. Med..

[47] Little P.J., Tannock L., Olin K.L., Chait A., Wight T.N. (2002). Proteoglycans synthesized by arterial smooth muscle cells in the presence of transforming growth factor-beta1 exhibit increased binding to LDLs. Arterioscler. Thromb. Vasc. Biol..

[48] Skalen K., Gustafsson M., Rydberg E.K., Hulten L.M., Wiklund O., Innerarity T.L., Boren J. (2002). Subendothelial retention of atherogenic lipoproteins in early atherosclerosis. Nature.

[49] Little P.J., Ballinger M.L., Burch M.L., Osman N. (2008). Biosynthesis of natural and hyperelongated chondroitin sulfate glycosaminoglycans: New insights into an elusive process. Open Biochem. J..

[50] Afroz R., Cao Y., Rostam M.A., Ta H., Xu S., Zheng W., Osman N., Kamato D., Little P.J. (2018). Signalling pathways regulating galactosaminoglycan synthesis and structure in vascular smooth muscle: Implications for lipoprotein binding and atherosclerosis. Pharmacol. Ther..

[51] Little P.J., Burch M.L., Getachew R., Al-aryahi S., Osman N. (2010). Endothelin-1 stimulation of proteoglycan synthesis in vascular smooth muscle is mediated by endothelin receptor transactivation of the transforming growth factor-[beta] type I receptor. J. Cardiovasc. Pharmacol..

[52] Afroz R., Zhou Y., Little P.J., Xu S., Mohamed R., Stow J., Kamato D. (2020). Toll-like Receptor 4 Stimulates Gene Expression via Smad2 Linker Region Phosphorylation in Vascular Smooth Muscle Cells. ACS Pharmacol. Transl. Sci..

[53] Rostam M.A., Shajimoon A., Kamato D., Mitra P., Piva T., Getachew R., Cao Y., Zheng W., Osman N., Little P.J. (2018). Flavopiridol inhibits TGF-beta-stimulated biglycan synthesis by blocking linker region phosphorylation and nuclear translocation of Smad2. J. Pharmacol. Exp. Ther..

[54] Mohamed R., Dayati P., Mehr R.N., Kamato D., Seif F., Babaahmadi-Rezaei H., Little P.J. (2019). Transforming growth factor-beta1 mediated CHST11 and CHSY1 mRNA expression is ROS dependent in vascular smooth muscle cells. J. Cell Commun. Signal..

[55] Kamato D., Do B.H., Osman N., Ross B.P., Mohamed R., Xu S., Little P.J. (2020). Smad linker region phosphorylation is a signalling pathway in its own right and not only a modulator of canonical TGF-beta signalling. Cell Mol. Life Sci..

[56] Kamato D., Ta H., Afroz R., Xu S., Osman N., Little P.J. (2019). Mechanisms of PAR-1 mediated kinase receptor transactivation: Smad linker region phosphorylation. J. Cell Commun. Signal..

[57] Kamato D., Burch M., Zhou Y., Mohamed R., Stow J.L., Osman N., Zheng W., Little P.J. (2019). Individual Smad2 linker region phosphorylation sites determine the expression of proteoglycan and glycosaminoglycan synthesizing genes. Cell Signal..

[58] Rostam M.A., Kamato D., Piva T.J., Zheng W., Little P.J., Osman N. (2016). The role of specific Smad linker region phosphorylation in TGF-beta mediated expression of glycosaminoglycan synthesizing enzymes in vascular smooth muscle. Cell Signal..

[59] Getachew R., Ballinger M.L., Burch M.L., Reid J.J., Khachigian L.M., Wight T.N., Little P.J., Osman N. (2010). PDGF beta-receptor kinase activity and ERK1/2 mediate glycosaminoglycan elongation on biglycan and increases binding to LDL. Endocrinology.

[60] Widlansky M.E., Gokce N., Keaney J.F., Vita J.A. (2003). The clinical implications of endothelial dysfunction. J. Am. Coll. Cardiol..

[61] Tak B.T., Balci K.G., Erken H., Gerede D.M., Tak S., Goksuluk H., Turhan S., Erol C. (2017). Evaluation of endothelial dysfunction with flow-mediated dilatation after transradial coronary angiography. Acta Cardiol..

[62] Mortensen S.P., Nyberg M., Thaning P., Saltin B., Hellsten Y. (2009). Adenosine contributes to blood flow regulation in the exercising human leg by increasing prostaglandin and nitric oxide formation. Hypertension.

[63] Anderson T.J., Uehata A., Gerhard M.D., Meredith I.T., Knab S., Delagrange D., Lieberman E.H., Ganz P., Creager M.A., Yeung A.C. (1995). Close relation of endothelial function in the human coronary and peripheral circulations. J. Am. Coll. Cardiol..

[64] Celermajer D.S., Sorensen K.E., Gooch V.M., Spiegelhalter D.J., Miller O.I., Sullivan I.D., Lloyd J.K., Deanfield J.E. (1992). Non-invasive detection of endothelial dysfunction in children and adults at risk of atherosclerosis. Lancet.

[65] Joannides R., Haefeli W.E., Linder L., Richard V., Bakkali E.H., Thuillez C., Luscher T.F. (1995). Nitric oxide is responsible for flow-dependent dilatation of human peripheral conduit arteries In Vivo. Circulation.

[66] Lieberman E.H., Gerhard M.D., Uehata A., Selwyn A.P., Ganz P., Yeung A.C., Creager M.A. (1996). Flow-induced vasodilation of the human brachial artery is impaired in patients <40 years of age with coronary artery disease. Am. J. Cardiol..

[67] Green D. (2005). Point: Flow-mediated dilation does reflect nitric oxide-mediated endothelial function. J. Appl. Physiol..

[68] Charakida M., Masi S., Luscher T.F., Kastelein J.J., Deanfield J.E. (2010). Assessment of atherosclerosis: The role of flow-mediated dilatation. Eur. Heart J..

[69] Halcox J.P., Donald A.E., Ellins E., Witte D.R., Shipley M.J., Brunner E.J., Marmot M.G., Deanfield J.E. (2009). Endothelial function predicts progression of carotid intima-media thickness. Circulation.

[70] Bailey T.G., Perissiou M., Windsor M.T., Schulze K., Nam M., Magee R., Leicht A.S., Green D.J., Greaves K., Golledge J. (2018). Effects of acute exercise on endothelial function in patients with abdominal aortic aneurysm. Am. J. Physiol. Heart Circ. Physiol..

[71] Grenon S.M., Chong K., Alley H., Nosova E., Gasper W., Hiramoto J., Boscardin W.J., Owens C.D. (2014). Walking disability in patients with peripheral artery disease is associated with arterial endothelial function. J. Vasc. Surg..

[72] Matsuzawa Y., Kwon T.G., Lennon R.J., Lerman L.O., Lerman A. (2015). Prognostic Value of Flow-Mediated Vasodilation in Brachial Artery and Fingertip Artery for Cardiovascular Events: A Systematic Review and Meta-Analysis. J. Am. Heart Assoc..

[73] Green D.J., Jones H., Thijssen D., Cable N.T., Atkinson G. (2011). Flow-mediated dilation and cardiovascular event prediction: Does nitric oxide matter?. Hypertension.

[74] Mortensen S.P., Askew C.D., Walker M., Nyberg M., Hellsten Y. (2012). The hyperaemic response to passive leg movement is dependent on nitric oxide: A new tool to evaluate endothelial nitric oxide function. J. Physiol..

[75] Trinity J.D., Groot H.J., Layec G., Rossman M.J., Ives S.J., Runnels S., Gmelch B., Bledsoe A., Richardson R.S. (2012). Nitric oxide and passive limb movement: A new approach to assess vascular function. J. Physiol..

[76] Bonetti P.O., Pumper G.M., Higano S.T., Holmes D.R., Kuvin J.T., Lerman A. (2004). Noninvasive identification of patients with early coronary atherosclerosis by assessment of digital reactive hyperemia. J. Am. Coll. Cardiol..

[77] Schnabel R.B., Schulz A., Wild P.S., Sinning C.R., Wilde S., Eleftheriadis M., Herkenhoff S., Zeller T., Lubos E., Lackner K.J. (2011). Noninvasive vascular function measurement in the community: Cross-sectional relations and comparison of methods. Circ. Cardiovasc. Imaging.

[78] Ioannidis J.P. (2009). Prediction of cardiovascular disease outcomes and established cardiovascular risk factors by genome-wide association markers. Circ. Cardiovasc. Genet..

[79] Holder S.M., Bruno R.M., Shkredova D.A., Dawson E.A., Jones H., Hopkins N.D., Hopman M.T.E., Bailey T.G., Coombes J.S., Askew C.D. (2021). Reference Intervals for Brachial Artery Flow-Mediated Dilation and the Relation with Cardiovascular Risk Factors. Hypertension.

